# Nanoparticulate Sodium Trimetaphosphate and Fluoride in Gels Affect Enamel Surface Free Energy After Erosive Challenge In Vitro

**DOI:** 10.3390/pharmaceutics17101356

**Published:** 2025-10-21

**Authors:** Beatriz Díaz-Fabregat, Alberto Carlos Botazzo Delbem, Wilmer Ramírez-Carmona, Letícia Cabrera Capalbo, Liliana Carolina Báez-Quintero, Caio Sampaio, Thayse Yumi Hosida, Douglas Roberto Monteiro, Juliano Pelim Pessan

**Affiliations:** 1 Department of Pediatric Dentistry and Public Health, School of Dentistry, Sao Paulo State University (UNESP), Araçatuba 16015-050, SP, Brazil; bdf930402@gmail.com (B.D.-F.); alberto.delbem@unesp.br (A.C.B.D.); wilmerramirezcarmona@gmail.com (W.R.-C.); lecapalbo@gmail.com (L.C.C.); lilianacarolina@gmail.com (L.C.B.-Q.); caio.sampaio@unesp.br (C.S.); thayse.hosida@unesp.br (T.Y.H.); douglas.monteiro@unesp.br (D.R.M.); 2 School of Dentistry, University of Western São Paulo (UNOESTE), Presidente Prudente 19026-310, SP, Brazil; 3 Postgraduate Program in Health Sciences, University of Western São Paulo (UNOESTE), Presidente Prudente 19026-310, SP, Brazil; 4 Division of Clinical Essentials and Simulation, University of Detroit Mercy, Detroit, MI 48221, USA; 5 Universidad Cooperativa de Colombia, Bogotá 111311, Colombia

**Keywords:** enamel, nanoparticles, polyphosphates, sodium fluoride, tooth erosion

## Abstract

**Background/Objectives**: The aim of this study was to evaluate the effects of sodium trimetaphosphate (TMP) and fluoride (F) on the surface free energy (SFE) of enamel coated with human salivary pellicle in vitro, both after treatment with the gels and after an erosive challenge. **Methods**: Bovine enamel discs (*n* = 10/group) were randomly allocated into seven treatment groups (gels): placebo (without any actives), low-fluoride gels (4500 ppm F—“4500F”) supplemented or not with microparticulate TMP (5%) or nanoparticulate (2.5% or 5%) TMP, 9000 ppm F (positive control), and 12,300 ppm F (acid gel, commercial control); a negative control group (i.e., untreated enamel) was included. Discs were exposed to human saliva (2 h), treated with the gels (1 min) and subjected to a 1-min acid challenge. Three probing liquids were used to assess enamel SFE. Data were submitted to two-way, repeated-measures ANOVA followed by Tukey’s test, and by Mann–Whitney’s test (*p* < 0.05). **Results**: SFE was significantly altered after exposure to saliva, changing from hydrophobic to slightly hydrophilic; gel treatment further increased enamel hydrophilicity (i.e., electron-donor properties), without significant differences among gels. After the erosive challenge, the enamel surface became significantly less hydrophilic for all groups; the highest values were observed for both gels containing nanoparticulate TMP. As for the overall SFE, the best performance was achieved by the gel containing 5% nanometric TMP. **Conclusions**: SFE of salivary-coated enamel was significantly influenced by the treatment gels, which promoted increases in hydrophilicity. Gels containing TMP, especially at nanoscale, promoted higher resistance to changes in hydrophilicity after an erosive challenge.

## 1. Introduction

Erosive tooth wear (ETW) has become an increasingly prevalent dental problem worldwide, due to its irreversible and cumulative nature, with varying effects on oral and dental health. Despite the effects of fluoride (F) on dental caries control being supported by a large number of well-designed clinical trials [1,2,3], limited scientific evidence is available to attest to its efficacy in protecting against ETW [4]. Indeed, calcium fluoride (CaF_2_) precipitates resulting from the application of F products are prone to dissolution under severe acid challenges, thereby providing limited protection against erosion [5]. This scenario has stimulated the search for alternative therapies to boost the effects of F against ETW.

In this context, the evidence attests to the synergistic action of sodium trimetaphosphate (TMP) and F in erosion models, when applied in vehicles for professional use or self-application [6,7,8,9,10,11]. Furthermore, such effects were shown to be further enhanced by the use of nanoparticles, due to their greater reactivity compared with conventional (i.e., micrometric) particles [12]. Considering F gels, the addition of TMP to low-F formulations (1% sodium fluoride—NaF, 4500 ppm F), either as microparticles or nanoparticles, resulted in a significantly higher protective effect against ETW compared with their TMP-free counterpart. However, only the gel supplemented with nano-sized TMP provided significantly lower ETW compared with a commercial acidic formulation (12,300 ppm F) [10].

Laboratory experiments with hydroxyapatite [8] and biofilms [13] provided some evidence on the mechanisms by which TMP and F act on the tooth structures (i.e., adsorption) and in the mineral dynamics (i.e., absorption). More recently, the evaluation of surface free energy (SFE) has been used as a tool to provide data on the physicochemical interactions between surfaces and ions or molecules from a given therapy [14,15,16]. Surface reactivity is evaluated by SFE from the contact angles formed by different probing liquids on a solid surface, and this property was shown to be closely linked to mineralization processes of the tooth structure in a study assessing the effects of F and sodium hexametaphosphate (HMP) [14]. Regarding TMP, two studies assessed changes in SFE of enamel [15] and dentin [16] after administering this polyphosphate, either alone or in combination with F, demonstrating that TMP significantly alters enamel surface properties and its ability to donate electrons, favoring cation attraction.

It is noteworthy, however, that the analysis of SFE is still not widely used in erosion models, so that further studies on the topic could provide additional data on its usefulness to better understand surface changes both after the application of a therapeutic agent and after an erosive challenge. Furthermore, one important limitation of the two aforementioned studies [15,16] is the absence of the acquired enamel pellicle, which is known to greatly affect the interactions between tooth surfaces and ions/molecules [17]. Also, both studies assessed the effects of TMP and F in aqueous solutions, which might result in different outcomes in comparison with more complex formulations, such as gels for professional applications. Finally, both studies assessed TMP administered as microparticles, so that the effects of nanometric TMP on enamel SFE remain unknown.

Based on these considerations, the present study aimed to evaluate the effects of gels containing TMP and F, alone or in combination, on the SFE of enamel coated with acquired salivary pellicle in vitro, both after gel treatment and following an erosive challenge. The null hypothesis tested was that SFE would not be affected by TMP, regardless of the particle size, either after gel treatment or erosive challenge.

## 2. Materials and Methods

### 2.1. Experimental Design, Ethical Aspects, and Sample Size Calculation

**Figure 1:**
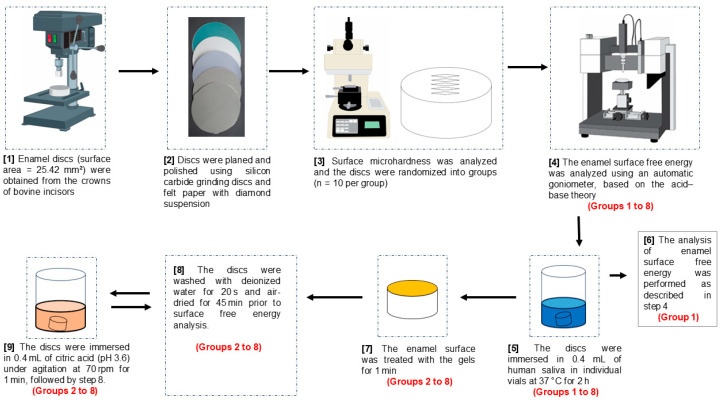
Schematic flowchart summarizing the experimental design of the study. Group 1: Negative control; Group 2: Placebo (gel without any actives), Group 3: low-fluoride gel (4500 ppm F, 4500F); Group 4: conventional neutral gel (9000 ppm F); Group 5: 4500F + 5% microparticulate TMP gel; Group 6: 4500F + 2.5% nanoparticulate TMP gel; Group 7: 4500F + 5% nanoparticulate TMP gel; and Group 8: 12,300 ppm F (acid gel).

Bovine enamel discs were selected by surface hardness, and randomly allocated into eight study groups (gels): Group 1: Negative control (i.e., untreated enamel); Group 2: Placebo (gel without active agents), Group 3: low-fluoride gel (4500 ppm F, “4500F”); Group 4: conventional neutral gel (9000 ppm F); Group 5: 4500F + 5% microparticulate TMP gel; Group 6: 4500F + 2.5% nanoparticulate TMP gel; Group 7: 4500F + 5% nanoparticulate TMP gel; and Group 8: 12,300 ppm F (acid gel). Discs were exposed to human saliva (2 h) to form acquired enamel pellicle, treated with the gels (1 min), and subjected to a 1-min acid challenge. Three probing liquids were used to assess enamel SFE using an automatic goniometer (DSA 100S, Krüss, Hamburg, Germany) at four different moments: prior to the beginning of the experiments (all 8 groups), after exposure to human saliva (negative control only), after treatment with the gels (all 7 test groups), and after an erosive challenge (all 7 test groups) (Figure 1). This in vitro study was approved by the Ethics Committee from São Paulo State University (Unesp), School of Dentistry, Araçatuba, Brazil, for experiments involving animals (Protocol 0486-2020) and humans (Protocol 67881223.5.0000.5420). A pilot study (*n* = 4/group) was performed to determine sample size, considering the minimum detectable mean difference between Groups 3 and 4. Mean difference (4.7 mN/m), standard deviation (2.6 mN/m), alpha (0.05), power (0.80), and the number of study groups (*n* = 7) were informed, resulting in a required sample size of ten discs per group.

### 2.2. Preparation of Enamel Discs

Bovine permanent incisors were collected and stored in 2% formaldehyde solution (pH 7.0) for 30 days, at room temperature [18]. The flattest portion of the vestibular surface of the crowns was used to obtain the enamel discs (Dinser, São Paulo, Brazil). The surfaces of the discs (area = 25.42 mm^2^) were flattened using a Beta Grinder-Polisher (Buehler, Lake Bluff, IL, USA), silicon carbide grinding discs (30-5108-400, -600, -800, and -012, Buehler), and felt paper (Polishing Cloth, 40-7618, Buehler) with diamond suspension (Extec Corp., Enfield, CT, USA) [11]. The discs were kept in an environment moistened with 2% formaldehyde solution at pH 7.0 [19].

Surface hardness was measured with a Micromet 5114 tester (Buehler, IL, USA) using a Knoop indenter (25 g, 10 s). Five central indentations (100 μm apart) were made per disc, and only specimens with 320–380 KHN were selected [19]. The mean (±standard deviation) values of microhardness were: 360.4 ± 14.8 (Group 1), 360.9 ± 13.7 (Group 2), 360.4 ± 13.6 (Group 3), 360.4 ± 13.4 (Group 4), 360.6 ± 13.2 (Group 5), 360.6 ± 13.0 (Group 6), 360.7 ± 12.8 (Group 7), and 360.8 ± 12.8 (Group 8).

### 2.3. Gel Formulation

All gels were manufactured (Department of Pediatric Dentistry and Public Health, School of Dentistry, Araçatuba, Sao Paulo State University, São Paulo, Brazil), following the protocol described by Díaz-Fabregat et al. [20] using the same concentrations tested in enamel erosion study [10], and contained: carboxymethylcellulose (Sigma-Aldrich Co., St Louis, MO, USA), sodium saccharin (Vetec, Duque de Caxias, Rio de Janeiro, Brazil), glycerol (Merck, Darmstadt, Germany), mint oil flavoring (Synth, Suzano, Brazil) and deionized water. The placebo gel (“PLA”) was produced without the addition of F or TMP. Sodium fluoride (NaF, Merck, Darmstadt, Germany) was used to prepare all gels containing 4500 or 9000 ppm F (positive control) (hereafter abbreviated as “4500F” and “9000F”). A gel containing 12,300 ppm F (“APF”, acid gel, DFL Dental Products, Rio de Janeiro, Brazil) was used as a commercial control. TMP microparticulate (average size of 450 ± 250 nm, 70 g, Na_3_O_9_P_3_, Aldrich, purity ≥ 95% CAS 7785-84-4, Sigma-Aldrich Co., St. Louis, MO, USA) at 5% and TMP nanoparticulate (approximately 22.7 nm, Chemistry Institute of the Federal University of São Carlos) at 2.5 or 5% were added to a 4500F gel, yielding three test formulations: TMPmicro5, TMPnano2.5, and TMPnano5, respectively. The nanoparticles synthesis and characterization were described in a previous study [21]. F concentrations and pH values were determined using the ion analyzer (Orion 720 A+; Orion Research Inc., San Francisco, CA, USA). Approximately 100 mg of each gel was dissolved in 100 mL of deionized water, in triplicate. Each solution was analyzed in duplicate to assess F concentrations, after buffering with TISAB II added at 1:1 proportion [22]. The pH was checked electrometrically for all gels [23]. Mean fluoride concentrations (Standard Deviation) in the PLA, 4500F, 9000F, TMPmicro5, TMPnano5, TMPnano2.5, and APF gels averaged 29.6 (1.2); 4215.8 (111.4); 8721.6 (141.6); 4736.1 (90.6); 4568.3 (97.3); 4071.6 (78.7); and 12,131.9 (126.3) ppm F, respectively. A neutral pH was determined for all gels (6.4, ranging from 6.3 to 6.7), except for the APF (pH = 3.6).

### 2.4. Stimulated Saliva Collection

Stimulated saliva samples were collected from healthy and non-smoking volunteers (*n* = 4). They were instructed to chew a piece of PARAFILM^®^ M (Sigma-Aldrich Co., St. Louis, MO, USA) and expectorate all saliva into ice-chilled vials. Collections were performed in the morning, at least 2 h after eating or drinking anything (except water) [24], and after thoroughly rinsing their mouth with water [25]. The stimulated saliva was pooled and centrifuged for 20 min at 4 °C and 4000× *g* [24]. The supernatant was collected, divided into eight 5 mL aliquots, and stored at −72 °C.

### 2.5. Surface Free Energy Analysis

Enamel SFE (γS, mN/m) was analyzed employing an automatic goniometer (DSA 100S, Krüss, Hamburg, Germany), using three probing liquids: diiodomethane, water and ethylene glycol. The enamel surface was divided into three regions; each liquid (0.5 μL) was automatically dispensed onto a different region using a glass syringe and a 0.5 mm gauge needle. Each drop was measured 5 times over 5 s at 23 °C and 44% ± 6 relative humidity [15,26,27]. The contact angles were measured from CCD-captured images using the tangent method (Drop Shape Analysis DSA4 Software, version 2.0–01, Krüss, Hamburg, Germany). The secondary parameters of γS included the acid-base interaction (γ^AB^; acid γ+, receptor component, and base γ−, donor component) and the Lifshitz van der Waals (γ^LW^, nonpolar component) contributions [26,27,28,29,30]. In addition, the free energy of interaction (ΔGsws, mJ m^−2^) between the surface (s) and the water (w) was calculated to determine the hydrophobicity (ΔGsws < 0) or hydrophilicity (ΔGsws > 0) of the enamel surface [27,28]. The ΔGsws^LW^ and ΔGsws^AB^ components were also assessed as secondary parameters of hydrophobicity/hydrophilicity parameters. All parameters were measured at 4 time points: prior to any exposure (*baseline*), after human’s saliva exposure (*pellicle*), after gel treatment exposure (*gel*), and after citric acid exposure (*erosion*).

### 2.6. Surface Treatment

The enamel surface was rinsed with deionized water for 20 s and air-dried for 45 min to obtain stable water contact angles [26]. The discs were initially evaluated (γS-*baseline*; ΔGsws-*baseline*) without any prior exposure. Next, all discs were immersed in 0.4 mL of human saliva in individual containers at 37 °C, unstirred, for 2 h to allow acquired enamel pellicle formation [9,31]. Thereafter, only the negative control group (i.e., untreated) was air-dried for 45 min and evaluated (γS-*pellicle* and ΔGsws-*pellicle*). All remaining discs (7 test groups) were gently blotted with a paper towel to remove excess saliva and received a single 1 min application of the assigned gel, which was then removed with deionized water for 20 s. Discs were then air-dried for 45 min prior to determining γS-*gel* and ΔGsws-*gel*. Finally, discs underwent an erosive challenge consisting of immersion in 1% citric acid (0.4 mL/disc, pH = 3.6, Synth, Suzano, Brazil) under agitation at 70 rpm for 1 min at room temperature, followed by rinsing with deionized water for 20 s [24,32]. Discs were again air-dried for 45 min, and γS-*erosion* and ΔGsws-*erosion* were assessed to complete the protocol.

### 2.7. Statistical Analysis

SFE (γS) and hydrophobicity/hydrophilicity (∆Gsws) were considered as the response variables, while treatment gels and enamel surface condition (baseline, gel, and erosion) were considered as the variation factors. To assess the effects of gels and surface conditions, the data were submitted to two-way repeated-measures ANOVA followed by Tukey’s post hoc test. Mann–Whitney’s test was used for the comparison between γS and ∆Gsws from the negative control group (γS-*baseline* and γS-*pellicle*). Analyses were performed using SigmaPlot software, version 12.0, with α = 0.05.

## 3. Results

**Figure 2:**
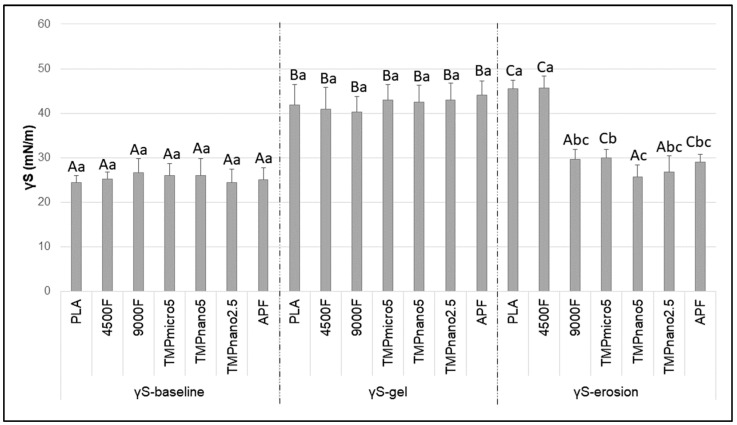
Mean surface free energy (γS, mN/m) according to the test groups, prior to any exposure (γS-*baseline*), after salivary exposure and treatment with the gels (γS-*gel*), and after citric acid exposure (γS-*erosion*). Bars denote standard deviations. Different letters indicate significant differences among the conditions of enamel surface within each group (upper-case) and among the groups within each condition of enamel surface (lower-case). Two-way ANOVA and Tukey’s test (*p* < 0.05; *n* = 10/group). Captions: PLA = placebo (with no actives); 4500F = 4500 ppm F; 9000F = 9000 ppm F; APF = 12,300 ppm F (acidulated); TMPmicro5 = 4500F + 5% micrometric TMP; TMPnano2.5 = 4500F + 2.5%TMP nanosized; TMPnano5 = 4500F + 5%TMP nanosized). TMP = sodium trimetaphosphate.

SFE (γS) was significantly influenced by the treatment gels (F = 19.9; *p* < 0.001) and by the enamel-surface condition (F = 511.1; *p* < 0.001), with significant interaction between the two factors (F = 29.2; *p* < 0.001). γS-*baseline* was lower than 30 mN/m for all groups, characteristic of hydrophobic surfaces. After treatment with the gels, γS-*gel* was significantly higher than γS-*baseline* (*p* < 0.05). After the erosive challenge (γS-*erosion*), groups treated with PLA, 4500F, TMPmicro5, and APF showed values significantly higher than their baseline counterparts (*p* < 0.05) (Figure 2). For the negative control group (untreated enamel), γS-*pellicle* (38.3 mN/m) was significantly higher than γS-*baseline* (26.2 mN/m) (*p* < 0.05), indicating a shift from hydrophobic to slightly hydrophilic behavior (∆Gsws-*baseline* = −3.6 mJ m^−2^, and ∆Gsws-*pellicle* = 1.2 mJ m^−2^; *p* = 0.910) (Supplementary Material, Table S1).

**Figure 3:**
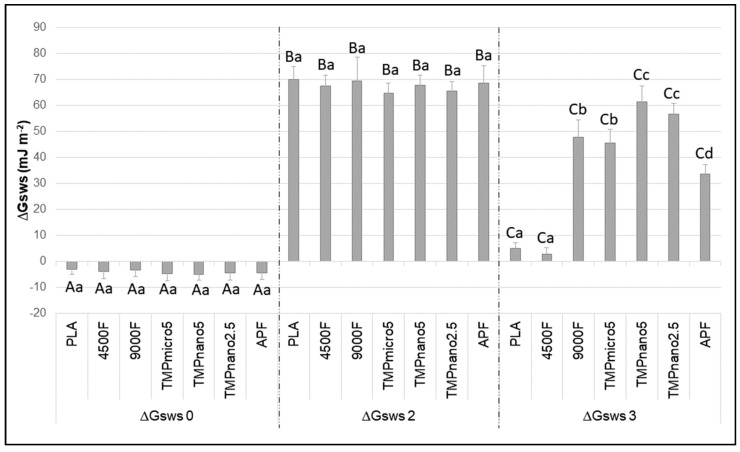
Mean of the hydrophobicity properties (ΔGsws, mJ m^−2^), according to the test groups, prior to any exposure (ΔGsws-*baseline*), after salivary exposure and treatment with the gels (ΔGsws-*gel*), and after citric acid exposure (ΔGsws-*erosion*). Bars denote standard deviations. Different letters indicate significant differences among the conditions of enamel surface within each group (upper-case) and among the groups within each condition of enamel surface (lower-case). Two-way ANOVA and Tukey’s test (*p* < 0.05, *n* = 10/group). Two-way ANOVA and Tukey’s test (*p* < 0.05; *n* = 10/group). Captions: PLA = placebo (with no actives); 4500F = 4500 ppm F; 9000F = 9000 ppm F; APF = 12,300 ppm F (acidulated); TMPmicro5 = 4500F + 5% micrometric TMP; TMPnano2.5 = 4500F + 2.5%TMP nanosized; TMPnano5 = 4500F + 5%TMP nanosized). TMP = sodium trimetaphosphate.

Consistent with the SFE data, surface hydrophobicity/hydrophilicity (∆Gsws) was significantly influenced by the treatment gels (F = 85.1; *p* < 0.001), and enamel surface condition (F = 4594.5; *p* < 0.001), with significant interactions between the two factors (F = 100.2; *p* < 0.001). At baseline, enamel surface exhibited hydrophobic properties (ΔGsws < 0), in contrast to the values obtained after saliva exposure and gel treatment (ΔGsws > 0), which yielded hydrophilic surfaces (*p* < 0.05) (Figure 3). After the erosive challenge (ΔGsws-*erosion*), the enamel surface became less hydrophilic than ΔGsws-*gel* for all groups (*p* < 0.05). Enamel treated with both gels containing nanoparticulate TMP (TMPNano2.5 and TMPnano5) exhibited the highest ΔGsws-*erosion* values, followed by 9000F, TMP5micro, and APF. The lowest values were observed for PLA and 4500F (*p* < 0.05) (Figure 3). Results of the secondary parameters contributing to the calculation of γS and ΔGsws are presented in the Supplementary Material.

## 4. Discussion

The increased incidence and prevalence of ETW in the primary dentition [33], along with the limited effects of conventional F therapies against tooth erosion, have encouraged the development of more effective and safe formulations for use in children. In this context, the addition of TMP to F vehicles for professional application has been shown to boost the effects of F against ETW, both in vitro and in situ [7,10,11]. In the present study, SFE was assessed to provide additional data on the mechanisms by which TMP and F act synergistically when administered in low-F gels (i.e., 4500F). Our findings demonstrated that SFE was significantly changed after acquired enamel pellicle formation, gel treatment and erosive challenge, with significant differences among the gels after acid exposure, thereby partially rejecting the study’s null hypothesis.

At baseline, SFE values indicated predominantly hydrophobic surfaces in polished specimens prior to saliva exposure, suggesting limited susceptibility to ion absorption, in line with data from previous studies employing the same methodology [14,15]. In those studies, exposure to aqueous cyclophosphate solutions (TMP or HMP) without F led to hydrophilic surfaces, favoring adsorption of Ca^2+^ and PO_4_^3−^ ions from the subsequent exposure to Ca^2+^/PO_4_^3−^ solutions. Although informative, these results represent direct TMP-enamel interactions under simplified conditions and may not fully replicate intraoral dynamics.

In contrast, in the present study, pellicle formation was allowed prior to any treatment, acknowledging the role of salivary proteins in modifying enamel surfaces and enabling ion and molecule binding from the oral environment [17,34]. Our data demonstrated that exposure to saliva significantly increased enamel SFE (from ~26 to ~38 mN/m), shifting the surface from hydrophobic to slightly hydrophilic, the latter being associated with greater mineral deposition compared with hydrophobic surfaces [14,15,16]. Interestingly, treatment with all gels promoted further increases in SFE, though to a smaller extent (average increment ~4 mN/m), suggesting that the pellicle itself exerted the greatest influence. These results emphasize the importance of including the salivary pellicle in experimental models assessing the effect of current or novel therapies intended to influence tooth mineral dynamics, to better simulate clinical conditions. Contrary to data on SFE, the effects of salivary pellicle on hydrophilicity were small (average increase of 4.8 mJ m^−2^) and not statistically significant, while treatment with the gels massively impacted enamel hydrophilicity (average increase of ~60 mJ m^−2^). This indicates that interpreting SFE and ∆Gsws together provides a more complete understanding of enamel surface changes. The lack of significant differences among the gels after application was somewhat unexpected based on previous data, suggesting that other gel components may have played a dominant role.

This interpretation is reinforced by the composition of the base gel, which included negatively charged compounds (e.g., carboxymethylcellulose and glycerol). These may have enriched enamel surfaces with electron-donor sites, increasing hydrophilicity independently of the active agents. Indeed, prior studies have shown that gels [30] or solutions [35] can alter the protein profile of the salivary pellicle, reinforcing the concept that non-active components may also influence changes in enamel SFE and ∆Gsws. Over time, non-active ingredients would be slowly washed out from tooth surfaces due to salivary clearance, so that any influence of the actives on enamel SFE and ∆Gsws might have been detected if time and clearance had been considered as variables in the present study.

The above-mentioned considerations are supported by the final set of results related to SFE and ∆Gsws after the erosive challenges. Overall, SFE significantly decreased for all groups treated with gels containing actives after the erosive challenges, except for 4500F, which behaved similarly to PLA, resulting in high enamel SFE (slightly higher than their counterparts after gel application). An opposite trend was observed for ∆Gsws, which plummeted after PLA and 4500F application, while decreases for the other gels were less pronounced. This suggests that citric acid exposure may have removed remnants of non-active compounds, revealing the effects of TMP and F on SFE and ∆Gsws.

The acid-base theory adopted here allows decomposition of SFE into polar and non-polar components [27,28]. For PLA and 4500F, post-erosion SFE values were greatly influenced by the non-polar component, which is related to aspects not involving electron-donor (γ−) or electron-receptor (γ+) properties (Supplementary Material, Table S1). The lesser influence of the polar component resulted in a surface with low ∆Gsws values, characteristic of low electron-donor potential, which does not facilitate the deposition of positively charged ions or molecules. Conversely, the remaining groups were mostly influenced by the polar (negative) component (γ−) [27,28]. As γ− is characteristic of high electron-donor potential, it could be expected that, in the oral environment, treatment with the TMP-containing gels would significantly enhance deposition of salivary minerals compared with, similarly with in vitro data on enamel Ca^2+^, PO_4_^3−^, F-uptake after treatment with the same gels of the current study [23].

The superior performance of TMP nanoparticles over microparticles when administered in F gels on ETW [10] provides further insights into the mechanisms involved. After the erosive challenge, both gels containing nanoparticulate TMP had significantly higher ∆Gsws values, indicating greater resistance to reductions in γ−, thus maintaining their high ability to retain positively charged ions or molecules. Reasons for the superior effect of nanoparticles have been extensively reported, mainly associated with their higher surface-to-volume ratio, which provides more reactive sites compared with microparticles [36]. This results in higher surface energy and reactivity, enhancing their ability to interact with enamel crystals. Notably, these changes in enamel SFE/hydrophilicity can be achieved using much lower nanoparticle concentrations than micrometric counterparts, due to the higher surface-to volume ratio. This may explain the lack of significant differences between the two nanosized TMP concentrations tested. It may be hypothesized that the greater electron availability and reactivity of nanoparticles were responsible for the increased resistance to γ− reductions after the erosive challenge, justifying the higher protective effect of low-fluoride gels containing nanometric TMP against ETW, even after intensive and cumulative erosive/abrasive challenges [10].

The increase in hydrophilicity (ΔGsws > 0) between gels with 4500F (low fluoride) and 9000F (high fluoride) at neutral pH reflects a significantly higher number of electron-donor sites in the high-fluoride gel. This likely results from increased fluoride on the enamel surface, enhancing the uptake of Ca^2+^ and PO_4_^3−^ ions from the surrounding environment [15]. As for the APF, its higher protective effect against ETW [10] was not associated with high ∆Gsws values after the erosive challenge in the present study. In fact, ∆Gsws values were the lowest among all gels containing active agents, except for 4500F. Considering that APF promotes the deposition of large amounts of CaF_2_, it is possible that Ca^2+^ ions released during the erosive challenge bound to enamel electron-donor sites resulting from the gel application, thus justifying the final ∆Gsws for the APF group. The enhanced enamel F uptake and CaF_2_ deposition promoted by high F levels under low pH may also explain the superior performance of APF compared with the other gels [23].

These considerations on mineral dynamics could be supported by data on enamel F, Ca, and P, as well as the ions released into the acid solution, which were not assessed in the present investigation, representing a study limitation. It should also be noted that enamel SFE assessment requires a dry surface, which may have altered pellicle properties through dehydration and changes in protein quaternary structure and functions. Thus, the results should be interpreted with caution, and future in vivo validation is needed. Within the limitations of this short-term, in vitro protocol, it can be concluded that enamel SFE is strongly influenced by the salivary pellicle. Therefore, protocols assessing different formulations on enamel mineral dynamics should account for this factor to better mimic clinical application. Also, the superior effect of TMP-containing formulations against ETW reported in the literature is associated with changes in enamel SFE, particularly ∆Gsws, by promoting surfaces with electron-donor properties. The higher reactivity of nanometric TMP was shown to enhance resistance in ∆Gsws changes (γ− reduction), providing further evidence of the mechanisms by which TMP, especially as nanoparticles, boosts the effect of low-F gels against ETW.

## 5. Conclusions

The SFE of salivary-coated enamel was significantly influenced by the treatment gels, which increased surface hydrophilicity. Gels containing TMP, particularly in nanopaticulate form, provided greater resistance to changes in hydrophilicity after an erosive challenge. These findings suggest that nanosized TMP enhances the protective effect of low-fluoride gels against ETW, supporting its potential as an effective additive in preventive formulations.

## 6. Patents

The second and the last author have a patent for a product used in the study, by the National Institute of Industrial Property—INPI/SP, on 11 April 2017, under number C1 0801811-1.

## Data Availability

Data may be available upon request to the corresponding author due to privacy/ethical restrictions.

## Supplementary Materials

The following supporting information can be downloaded at: https://www.mdpi.com/article/10.3390/pharmaceutics17101356/s1, Table S1: Mean (Standard Deviation) of all parameters adopted to calculate enamel surface free energy (γS, mN/m) and hydrophobicity/hydrophilicity.

## Abbreviations

The following abbreviations are used in this manuscript:

TMPnano2.5	TMP nanoparticulate at 2.5% + 4500 ppm F
4500F	4500 ppm F
TMPmicro5	TMP microparticulate at 5% + 4500 ppm F
TMPnano5	TMP nanoparticulate at 5% + 4500 ppm F
9000F	9000 ppm F
APF	Acidulated phosphate fluoride
*baseline*	Aferição ou análise nos espécimes previamente ao início do protocolo experimental
ETW	Erosive tooth wear
F	Fluoride
NaF	Sodium fluoride
PLA	Placebo
SFE/γS	Surface free energy
TMP	Sodium trimetaphosphate
γ−	Donor component (base)
γ+	Receptor component (acid)
γ^AB^	Acid base interaction, polar component
γ^LW^	Surface tension Lifshiz van der Waals, nonpolar component
ΔGsws	Free energy of interaction between the surface (s) and the water (w)
ΔGsws^AB^	Free energy of interaction between the surface (s) and the water (w) for polar component.
ΔGsws^LW^	Free energy of interaction between the surface (s) and the water (w) for nonpolar component
