# Supplementary material for: Nanoparticulate Sodium Trimetaphosphate and Fluoride in Gels Affect Enamel Surface Free Energy After Erosive Challenge In Vitro
Source: Pharmaceutics. 2025 Oct 21;17(10):1356. doi: 10.3390/pharmaceutics17101356 (PMC12567421; doi:10.3390/pharmaceutics17101356)
Supplement: Supplementary file 1 [file pharmaceutics-17-01356-s001.zip › pharmaceutics-3848183-supplementary.pdf]

Nanoparticulate Sodium Trimetaphosphate and Fluoride in Gels Affect Enamel Surface Free Energy after Erosive Challenge In Vitro

**Beatriz Díaz-Fabregat [1,2], Alberto Carlos Botazzo Delbem [1], Wilmer Ramírez-Carmona [1,3], Letícia Cabrera Capalbo [1,4], Liliana Carolina Báez-Quintero [1,5], Caio Sampaio [1], Thayse Yumi Hosida [1], Douglas Roberto Monteiro [1]
and Juliano Pelim Pessan [1,*]**

Mean (Standard Deviation) of all parameters adopted to calculate enamel surface free energy (γS, mN/m) and hydrophobicity/hydrophilicity

		Water (°)	*Diido-methane* (°)	*Ethylene Glycol* (°)	**s (mN/m)**	**s^{LW} (mN/m)**	**s^{AB} (mN/m)**	**$s+$ (mN/m)**	**$s-$ (mN/m)**	**G_{sws}^{LW}**	**G_{sws}^{AB}**	**G_{sws}^{TOTAL}**
baseline	**Untreated**	75.6 (21.1)	59.0 (6.7)	63.4 (14.1)	26.2 (1.8) ^{Aa}	29.0 (1.7) ^{Aa}	-2.8 (1.6) ^{Aa}	0.1 (0.2) ^{Aa}	24.2 (2.0) ^{Aa}	-1.1 (0.4) ^{Aa}	-2.6 (4.0) ^{Aa}	-3.6 (3.9) ^{Aa}
	**PLA**	73.4 (20.3)	60.9 (4.3)	59.4 (10.8)	24.3 (1.6) ^{Aa}	27.4 (1.5) ^{Aa}	-3.3 (1.2) ^{Aa}	0.3 (0.3) ^{Aa}	24.3 (1.1) ^{Aa}	-0.7 (0.3) ^{Aa}	-2.2 (2.0) ^{Aa}	-2.9 (2.1) ^{Aa}
	**4500F**	73.3 (19.8)	60.1 (3.8)	62.2 (8.4)	25.1 (1.6) ^{Aa}	28.9 (1.5) ^{Aa}	-3.8 (1.4) ^{Aa}	0.3 (0.4) ^{Aa}	24.0 (1.3) ^{Aa}	-1.0 (0.4) ^{Aa}	-2.8 (2.7) ^{Aa}	-3.9 (2.6) ^{Aa}
	**9000F**	72.8 (19.7)	59.9 (4.2)	60.9 (11.1)	26.6 (3.1) ^{Aa}	29.2 (2.2) ^{Aa}	-3.2 (1.5) ^{Aa}	0.2 (0.1) ^{Aa}	24.3 (1.2) ^{Aa}	-1.2 (0.6) ^{Aa}	-2.3 (2.3) ^{Aa}	-3.4 (2.4) ^{Aa}
	**TMPmicro5**	77.2 (21.6)	59.9 (5.4)	61.1 (16.7)	26.0 (2.7) ^{Aa}	29.7 (2.1) ^{Aa}	-4.0 (2.8) ^{Aa}	0.2 (0.2) ^{Aa}	23.7 (1.3) ^{Aa}	-1.3 (0.6) ^{Aa}	-3.3 (2.6) ^{Aa}	-4.6 (2.9) ^{Aa}
	**TMPnano5**	69.2 (21.0)	59.8 (5.4)	59.8 (14.8)	26.0 (3.8) ^{Aa}	29.2 (2.7) ^{Aa}	-4.0 (1.9) ^{Aa}	0.3 (0.3) ^{Aa}	23.5 (1.1) ^{Aa}	-1.2 (0.8) ^{Aa}	-3.7 (2.1) ^{Aa}	-4.9 (2.2) ^{Aa}
	**TMPnano2.5**	74.1 (18.6)	60.3 (6.2)	63.4 (14.0)	24.4 (3.0) ^{Aa}	28.8 (2.1) ^{Aa}	-4.5 (1.9) ^{Aa}	0.3 (0.2) ^{Aa}	23.7 (1.4) ^{Aa}	-1.0 (0.6) ^{Aa}	-3.4 (2.6) ^{Aa}	-4.4 (2.8) ^{Aa}
	**APF**	74.4 (16.6)	61.5 (4.3)	64.2 (9.8)	25.0 (2.7) ^{Aa}	28.4 (2.0) ^{Aa}	-3.4 (2.0) ^{Aa}	0.4 (0.4) ^{Aa}	23.5 (1.3) ^{Aa}	-0.9 (0.5) ^{Aa}	-3.6 (2.3) ^{Aa}	-4.5 (2.3) ^{Aa}
pellicle	**Untreated**	54.3 (14.2)	60.3 (5.9)	34.5 (11.2)	38.3 (3.3)*	27.9 (4.3)	10.7 (2.4)*	2.3 (1.7)	27.8 (18.8)	-1.0 (1.2)	2.2 (27.0)	1.2 (26.3)
gel	**PLA**	24.7 (14.7)	50.9 (5.6)	23.3 (11.1)	41.8 (4.7) ^{Ba}	14.5 (2.7) ^{Bab}	28.3 (4.5) ^{Bab}	0.1 (0.1) ^{Aa}	76.0 (3.9) ^{Babc}	-1.7 (1.0) ^{Aab}	71.5 (4.2) ^{Ba}	69.8 (5.0) ^{Ba}
	**4500F**	22.7 (5.8)	75.0 (19.6)	31.4 (10.6)	40.9 (4.9) ^{Ba}	14.6 (4.2) ^{Bab}	26.3 (5.6) ^{Bab}	0.1 (0.1) ^{Aa}	76.2 (4.5) ^{Babc}	-2.1 (1.6) ^{Aab}	69.6 (4.5) ^{Ba}	67.5 (4.2) ^{Ba}
	**9000F**	27.8 (5.1)	88.0 (9.9)	39.6 (5.2)	40.3 (3.4) ^{Ba}	14.3 (2.6) ^{Bab}	27.4 (4.5) ^{Bab}	0.3 (0.4) ^{ABa}	79.7 (7.2) ^{Ba}	-1.8 (1.1) ^{Aab}	71.1 (8.7) ^{Ba}	69.3 (9.2) ^{Ba}
	**TMPmicro5**	23.2 (9.4)	81.0 (16.8)	27.1 (5.4)	43.0 (3.5) ^{Ba}	15.8 (4.6) ^{Babc}	28.2 (2.7) ^{Bab}	0.1 (0.1) ^{Aa}	73.6 (2.6) ^{Bbc}	-1.7 (1.5) ^{Aab}	66.3 (3.9) ^{Ba}	64.6 (4.0) ^{Ba}
	**TMPnano5**	24.2 (5.1)	82.9 (15.8)	25.8 (7.2)	42.5 (3.7) ^{Ba}	12.8 (2.8) ^{Ba}	30.4 (3.2) ^{Ba}	0.1 (0.1) ^{Aa}	75.8 (3.1) ^{Babc}	-2.7 (1.6) ^{Bb}	70.5 (3.2) ^{Ba}	67.7 (3.8) ^{Ba}
	**TMPnano2.5**	30.5 (9.6)	83.6 (15.2)	28.9 (6.6)	43.0 (3.8) ^{Ba}	16.9 (3.4) ^{Bbc}	26.1 (4.1) ^{Bb}	0.2 (0.1) ^{Aa}	74.5 (3.3) ^{Bbc}	-1.0 (1.2) ^{Aa}	66.5 (3.6) ^{Ba}	65.5 (3.5) ^{Ba}
	**APF**	27.7 (8.8)	60.3 (17.9)	32.5 (8.1)	44.0 (3.3) ^{Ba}	18.4 (3.3) ^{Bc}	26.0 (4.1) ^{Bb}	0.3 (0.3) ^{ABa}	77.2 (4.2) ^{Babc}	-0.6 (0.6) ^{Aa}	69.2 (6.7) ^{Ba}	68.6 (6.6) ^{Ba}

erosion	**PLA**	50.6 (8.1)	18.6 (5.1)	28.8 (17.7)	45.5 (1.9) ^{Ca}	48.4 (1.5) ^{Ca}	-2.8 (1.8) ^{Aa}	0.1 (0.1) ^{Aa}	34.4 (1.3) ^{Ca}	-10.5 (1.0) ^{Ba}	15.6 (2.0) ^{Ca}	5.1 (2.2) ^{Ca}
	**4500F**	52.3 (12.9)	21.9 (10.8)	20.5 (6.1)	45.7 (2.6) ^{Ca}	48.7 (1.4) ^{Ca}	-3.1 (2.5) ^{Aa}	0.1 (0.1) ^{Aa}	33.2 (2.0) ^{Ca}	-10.7 (0.9) ^{Ba}	13.5 (3.1) ^{Ca}	2.9 (2.5) ^{Ca}
	**9000F**	31.3 (15.1)	54.7 (17.8)	34.5 (9.3)	29.6 (2.3) ^{Abc}	39.7 (4.0) ^{Cbcd}	-10.1 (3.1) ^{Cb}	0.4 (0.2) ^{Bb}	65.4 (7.8) ^{Cbc}	-5.4 (2.0) ^{Bbc}	53.2 (7.8) ^{Cb}	47.7 (6.8) ^{Cb}
	**TMPmicro5**	40.3 (19.3)	46.7 (15.7)	37.9 (14.9)	29.9 (2.0) ^{Cb}	41.0 (4.1) ^{Cde}	-12.1 (3.9) ^{Cb}	0.2 (0.3) ^{Aab}	61.0 (5.5) ^{Cc}	-6.1 (2.1) ^{Bb}	51.5 (4.9) ^{Cb}	45.4 (5.3) ^{Cb}
	**TMPnano5**	32.8 (11.9)	47.2 (10.0)	32.5 (9.3)	25.7 (2.7) ^{Ac}	36.9 (2.8) ^{Ccfg}	-12.2 (4.7) ^{Cb}	0.1 (0.2) ^{Aa}	71.7 (4.6) ^{Cd}	-4.0 (1.3) ^{Ccd}	65.2 (6.5) ^{Cc}	61.2 (6.2) ^{Cc}
	**TMPnano2.5**	46.2 (17.9)	47.1 (10.2)	39.3 (9.7)	26.8 (3.6) ^{Abc}	37.8 (2.4) ^{Cbeg}	-10.9 (4.1) ^{Cb}	0.1 (0.1) ^{Aa}	67.8 (4.2) ^{Cbd}	-4.4 (1.2) ^{Bc}	61.0 (4.1) ^{Cc}	56.6 (4.2) ^{Cc}
	**APF**	46.8 (5.3)	44.3 (7.5)	39.5 (7.6)	29.0 (1.8) ^{Cbc}	34.0 (1.3) ^{Cf}	-5.0 (2.0) ^{Aa}	0.1 (0.1) ^{Ba}	48.5 (3.7) ^{Ce}	-2.7 (0.5) ^{Bd}	36.2 (3.6) ^{Cd}	33.5 (3.6) ^{Cd}

Table S1. Mean of secondary parameters assessed according to the test groups, prior to any exposure (*baseline*), after salivary exposure (*pellicle*) and treatment with the gels (*gel*), and after citric acid exposure (*erosion*). Bars denote standard deviations. Different letters indicate significant differences among the conditions of enamel surface within each group (upper-case) and among the groups within each condition of enamel surface (lower-case). *Different between *baseline* and *pellicle* in untreated group. Two-way ANOVA and Tukey's test, and by Mann-Whitney's test ($p < 0.05$, $n = 10$/group). Captions: = contact angle; Untreated = negative control group; PLA = placebo (with no actives); 4500F = 4,500 ppm F; 9000F = 9,000 ppm F; APF = 12,300 ppm F (acidulated); TMPmicro5 = 4500F + 5% micrometric TMP; TMPnano2.5 = 4500F + 2.5%TMP nanosized; TMPnano5 = 4500F + 5%TMP nanosized). TMP = sodium trimetaphosphate. γ^{LW}: surface tension Lifshiz van der Waals, nonpolar component; γ^{AB}: acid base interaction, polar component; $\gamma+$: receptor component (acid); $\gamma-$: donor component (base); $\Delta Gsws^{LW}$: free energy of interaction between the surface (s) and the water (w) for nonpolar component; and $\Delta Gsws^{AB}$: free energy of interaction between the surface (s) and the water (w) for polar component.
